# Long non-coding RNA UCA1 promotes gallbladder cancer progression by epigenetically repressing p21 and E-cadherin expression

**DOI:** 10.18632/oncotarget.18204

**Published:** 2017-05-25

**Authors:** Qiang Cai, Longyang Jin, Shouhua Wang, Di Zhou, Jiandong Wang, Zhaohui Tang, Zhiwei Quan

**Affiliations:** ^1^ Department of General Surgery, XinHua Hospital, Shanghai JiaoTong University School of Medicine, Shanghai 200092, China

**Keywords:** lncRNA UCA1, gallbladder cancer, EZH2, p21, E-cadherin

## Abstract

A growing number of studies indicated that long non-coding RNAs (lncRNAs) determine some cellular processes in cancer, such as proliferation, metastasis and differentiation. Urothelial carcinoma associated 1 (UCA1), an lncRNA, had been reported for its overexpression and oncogenic effect on various human cancers. In this study, we found that UCA1 was significantly overexpressed in gallbladder cancer (GBC) and positively correlated with tumor size, lymph node metastasis, TNM stage and short survival time. Moreover, UCA1 promoted GBC cell proliferation and metastasis *in vitro* and tumor growth *in vivo*. Mechanically, we identified that UCA1 promoted GBC progression through recruiting enhancer of zeste homolog 2 (EZH2) to the promoter of p21 and E-cadherin, and epigenetically suppressing their transcript.

## INTRODUCTION

Gallbladder cancer (GBC) is the fifth most highly diagnosed type of gastrointestinal tract malignancy and the most common bile duct malignancy [1, 2]. GBC is highly aggressive and spreads to regional lymph nodes at early stage [3]. In addition, it has a high rate of recurrence and chemo-resistance. Patients with GBC generally have a poor prognosis: the overall 5-year survival is less than 5%, the mean survival is 4-6 months [4]. Although massive efforts have been made to develop the effective therapy for GBC, however, little success has been achieved in reducing the mortality rates [5]. Therefore, improved insight into the precise molecular mechanisms of GBC progression is urgent to figure out the way to the diagnosis and treatment for GBC.

Long non-coding RNAs (lncRNAs) are a class of non-coding transcripts longer than 200 nucleotides. LncRNAs could regulate the expression of target genes via various mechanisms, like functioning as competing endogenous RNAs, serving as scaffolds or recruiting chromatin-modifying enzymes to target genes [6–8]. Mounting evidence revealed that the dysregulation of lncRNAs might be associated to the misbehavior of cancer progression. In our previous studies, lncRNAs GCASPC, MINCR, CCAT1 and LINC00152 play multi-roles including oncogenes or tumor suppressors in GBC [6, 9–12]. Urothelial carcinoma associated 1 (UCA1), an lncRNA that maps to chromosome 19p13.12, was initially identified in human bladder transitional cell carcinoma [13]. The ‘sponge’ role of UCA1 in cytoplasm had been investigated more than once. Through binding to microRNAs (miRNAs) response elements that could directly suppress their target protein expression, UCA1 has an oncogenic function on various human cancers, such as bladder cancer [14], non-small cell lung cancer [15], hepatocellular carcinoma [16] and etc. Hu et al. [17] also reported that UCA1 was physically associated with enhancer of zeste homolog 2 (EZH2), which suppressed p27 (Kip) through histone methylation (H3K27me3) on p27 (Kip) promoter in nucleus. However, little was known about the expression level of UCA1 and its underlying molecular mechanisms in cell proliferation and metastasis in GBC.

In the present study, we demonstrated the novel role of UCA1 in GBC and figured out that: a) UCA1 was upregulated in GBC; b) the upregulation of UCA1 was related to the tumor size, lymph node metastasis, TNM stage and overall survival of GBC patients; c) UCA1 promoted GBC cell proliferation and metastasis; d) UCA1 promoted tumorigenicity in nude mice; e) UCA1 played a pivotal role in GBC cell proliferation and metastasis through epigenetically regulating the expression of p21 and E-cadherin by interacting with EZH2.

## RESULTS

### High expression of UCA1 was associated with tumor size, lymph node metastasis, TNM stage and short survival time in GBC patients

**Figure 1:**
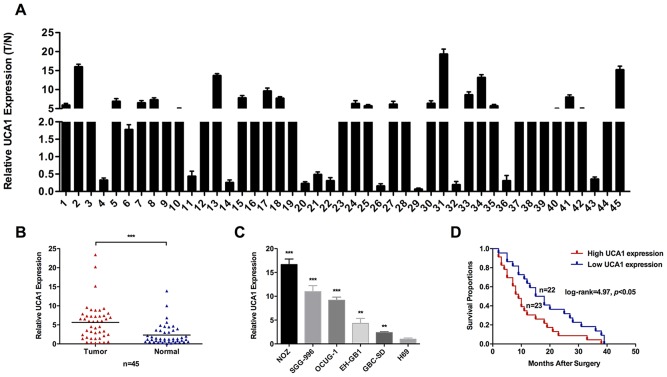
Relative expression of UCA1 in GBC and its clinical significance **(A** and **B)** Relative expression of UCA1 in GBC tissues and neighboring noncancerous tissues was detected by qRT-PCR (n = 45). Relative expression of UCA1 was normalized to GAPDH. **(C)** Relative expression of UCA1 in GBC cell lines (EH-GB1, GBC-SD, NOZ, OCUG-1 and SGC-996) and human gallbladder epithelium cell line H69 was detected by qRT-PCR. **(D)** Kaplan–Meier method with the log-rank test was used to analyze the overall survival curves of patients in high and low UCA1 expression groups (log-rank=4.97, *p*<0.05). The mean ± SD of triplicate experiments were plotted, **p<0.01, ***p<0.001.

To explore whether the expression of UCA1 was elevated in GBC progression, we first detected UCA1 expression levels in forty-five pairs of GBC tissues and neighboring noncancerous tissues by qRT-PCR. As shown in Figure 1A and 1B, the transcript levels of UCA1 were significantly higher in GBC tissues compared with neighboring noncancerous tissues from the same patient. Then, we detected UCA1 expression levels in four human GBC cell lines and human non-tumorigenic biliary epithelial cell line H69. The transcript levels of UCA1 were generally higher in GBC cell lines compared with H69 (Figure 1C). These results suggested that UCA1 was a high expression lncRNA in GBC.

To further explore the correlation between the transcript levels of UCA1 and GBC patients’ clinical and pathologic characteristics, we divided forty-five GBC patients into high UCA1 expression group (n=23, UCA1 expression ratio ≥ median ratio) and low UCA1 expression group (n=22, UCA1 expression ratio ≤ median ratio). Statistical analysis indicated that UCA1 expression was significantly associated with tumor size, lymph node metastasis, and TNM stage (Table 1). However, UCA1 expression was not associated with gender, age, local invasion or histological grade. Furthermore, Kaplan-Meier analysis indicated that patients with higher UCA1 expression levels might have a shorter survival time than those with lower levels (Figure 1D).

**Table 1:** The association of UCA1 expression in 45 GBC patients with clinicopathologic charateristics

Characteristics	Case number	UCA1 expression		*p*-Value
		Low (n=22)	High (n=23)	
Gender				0.445
Male	12	7	5	
Female	33	15	18	
Age				0.436
≤ 60	26	14	12	
> 60	19	8	11	
Tumor size				0.047*
≤ 5cm	26	16	10	
> 5cm	19	6	13	
Local invasion				0.256
Yes	29	16	13	
No	16	6	10	
Lymph node metastasis				0.035*
Yes	30	18	12	
No	15	4	11	
Histological grade				0.586
well and morderately	31	16	15	
Poorly and others	14	6	8	
TNM stage				0.042*
I-II	14	10	4	
III-IV	31	12	19	

* *P*<0.05.

### UCA1 promoted GBC cell proliferation *in vitro* and *in vivo*

**Figure 2:**
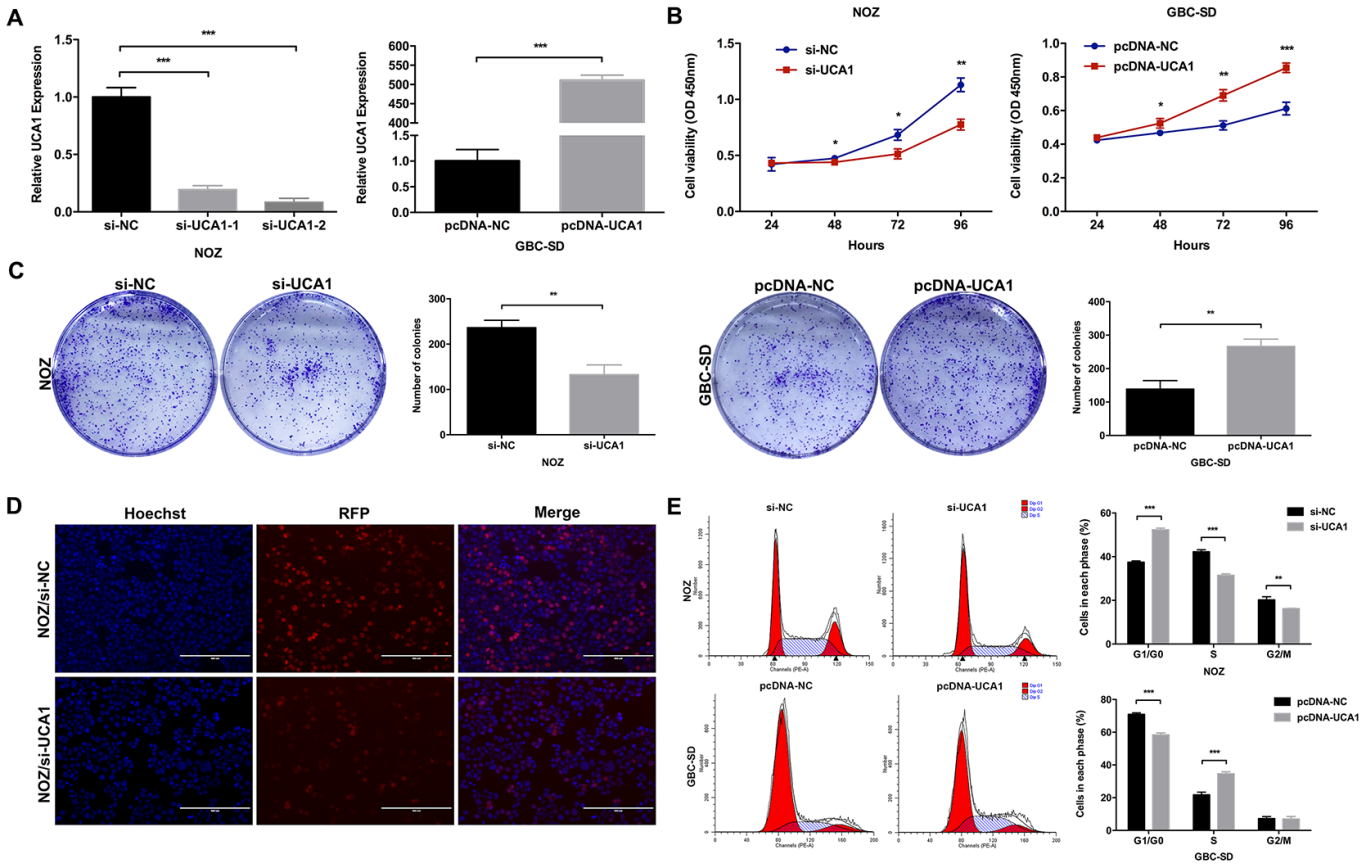
Effect of UCA1 on GBC cell growth *in vitro* **(A)** Relative expression of UCA1 in si-UCA1-transfected NOZ cells, or pcDNA-UCA1-transfected GBC-SD cells was detected by qRT-PCR. **(B)** The cell viability of si-UCA1-transfected NOZ cells, or pcDNA-UCA1-transfected GBC-SD cells was determined by CCK8 assays. **(C)** The coloning ability of si-UCA1-transfected NOZ cells, or pcDNA-UCA1-transfected GBC-SD cells was determined by colony formation assays. **(D)** The cell viability of si-UCA1-transfected NOZ cells was determined by EdU retention assay. **(E)** Flow cytometric analyses were performed to determine the cell cycle progression in si-UCA1-transfected NOZ cells, or pcDNA-UCA1-transfected GBC-SD cells. The mean ± SD of triplicate experiments were plotted, *p<0.05, **p<0.01, ***p<0.001.

To explore whether UCA1 was functionally involved in GBC cell proliferation, we designed two different UCA1 siRNAs to transfect NOZ cells that presented relatively high expression level of UCA1. Then we selected si-UCA1-2 for the subsequent experiments for its higher efficiency (Figure 2A). While, we also established stable UCA1-overexpression GBC-SD cell line that presented relatively low expression level of UCA1 by transfecting pcDNA-UCA1 (Figure 2A). Cell Counting Kit-8 (CCK8) and colony formation assays were performed to evaluate whether UCA1 indeed participated in GBC cell proliferation *in vitro*. As shown in Figure 2B and 2C, UCA1 knockdown significantly inhibited NOZ cells proliferation, while UCA1 overexpression significantly promoted GBC-SD cells proliferation. Furthermore, EdU retention assay confirmed the inhibitory effect of UCA1 knockdown in NOZ cells proliferation (Figure 2D).

To investigate whether the promotion of UCA1 on GBC cell proliferation *in vitro* was due to its regulation on cell cycle or apoptosis, we performed the flow cytometric analysis and found that UCA1 knockdown led to a significant G1-phase arrest of NOZ cells and UCA1 overexpression promoted GBC-SD cells cycle progression (Figure 2E). These results suggested that UCA1 was functionally involved in the regulation of G1/S cell-cycle transition in GBC cells. However, we did not observe any difference in the apoptotic rate between different groups (data not shown).

**Figure 3:**
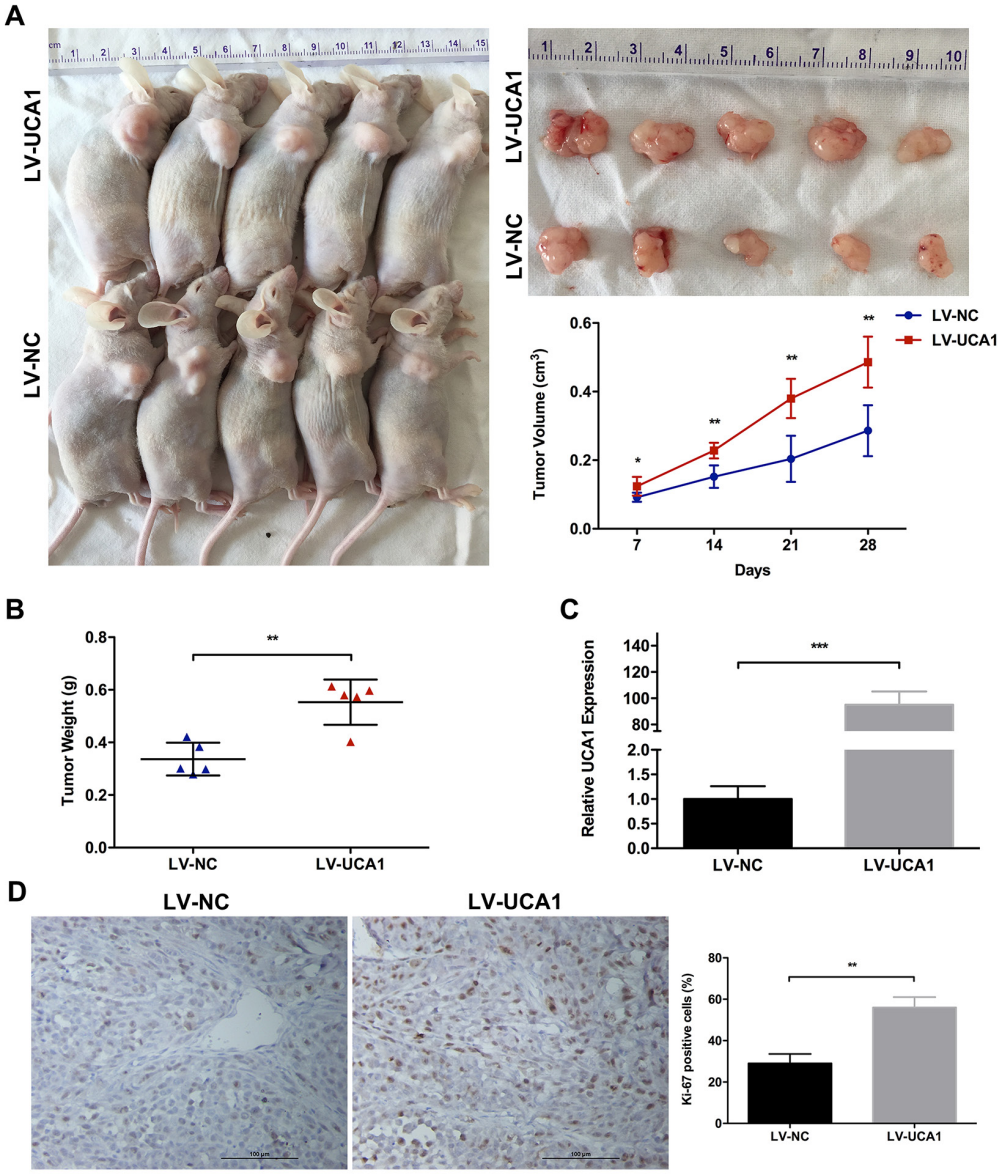
Effect of UCA1 overexpression on tumor growth *in vivo* **(A)** The nude mice carrying tumors from GBC-SD/LV-UCA1 and GBC-SD/LV-NC groups were shown. Tumor growth curves were calculated per week. **(B)** Tumor weight from GBC-SD/LV-UCA1 and GBC-SD/LV-NC groups was shown. **(C)** Relative expression of UCA1 in tumors from GBC-SD/LV-UCA1 and GBC-SD/LV-NC groups was detected by qRT-PCR. **(D)** The Ki-67 expression and positive cell numbers was determined by immunohistochemical staining. The mean ± SD of triplicate experiments were plotted, *p<0.05, **p<0.01, ***p<0.001.

To confirm the results from the *in vitro* studies, we then compared the tumorigenesis of GBC cell *in vivo*. GBC-SD cells, transfected with either LV-NC or LV-UCA1, were subcutaneously injected into nude mice. In comparison, tumor developed obviously faster in LV-UCA1 group than that in LV-NC group (Figure 3A). Meanwhile, the tumor weight was significantly heavier in LV-UCA1 group than that in LV-NC group (Figure 3B). In addition, we performed qRT-PCR in mouse tumor tissues and confirmed the significance of UCA1 overexpression in LV-UCA1 group (Figure 3C). Moreover, immunohistochemical staining showed that increased proliferative index Ki-67 expression in LV-UCA1 infected tumor tissues (Figure 3D). These results together suggested that UCA1 could promote GBC cell proliferation *in vitro* and *in vivo*.

### UCA1 promotes GBC cell metastasis and epithelial-mesenchymal transition (EMT) progression

**Figure 4:**
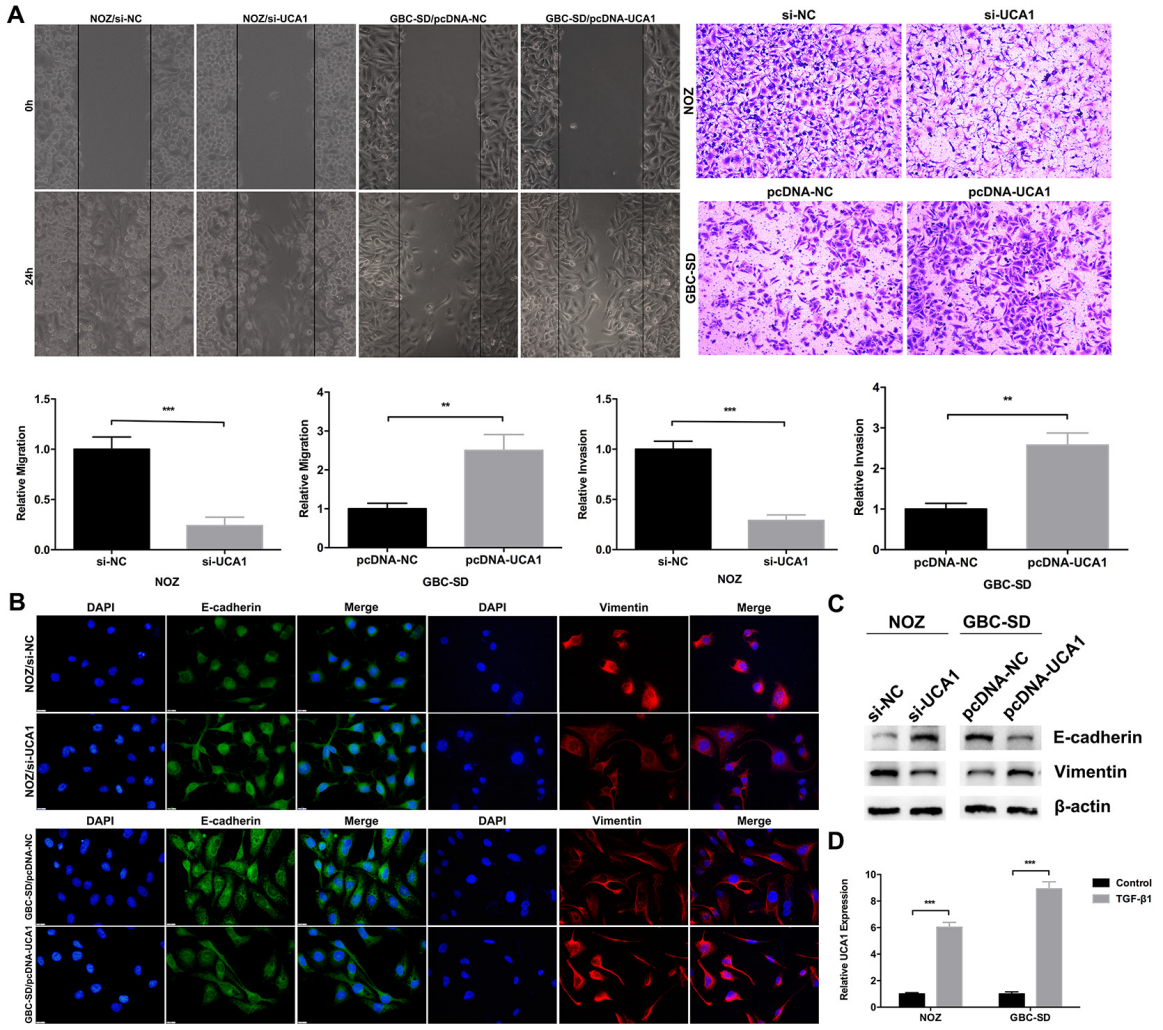
Effect of UCA1 on GBC cell metastasis and EMT *in vitro* **(A)** The cell migration and invasion ability of si-UCA1-transfected NOZ cells, or pcDNA-UCA1-transfected GBC-SD cells were determined by wound healing and transwell invasion assays, respectively. **(B** and **C)** The protein levels of E-cadherin and Vimentin in si-UCA1-transfected NOZ cells, or pcDNA-UCA1-transfected GBC-SD cells were determined by immunofluorescenceand western blot assays. **(D)** Relative expression of UCA1 in TGF-β1 treated NOZ and GBC-SD cells was detected by qRT-PCR. The mean ± SD of triplicate experiments were plotted, **p<0.01, ***p<0.001.

To determine the molecular function of UCA1 in GBC cell metastasis, wound healing and transwell invasion assays were performed to evaluate the ability of GBC cell migration and invasion. Compared with the control, UCA1 knockdown significantly inhibited NOZ cells migration and invasion, while UCA1 overexpression significantly promoted GBC-SD cells migration and invasion (Figure 4A). Accumulating evidence had validated that EMT, a process endowing epithelial cells with mesenchymal properties, plays an important role in GBC [12, 18–20]. Next, we performed immunofluorescence and western blot assays and revealed that UCA1 knockdown increased the epithelial marker E-cadherin expression and decreased the mesenchymal marker Vimentin expression in NOZ cells (Figure 4B and 4C). While, the opposite phenomenon was observed after UCA1 was overexpressed in GBC-SD cells (Figure 4B and 4C). Considered that we had successfully established EMT model in NOZ and GBC-SD cell lines by TGF-β1 [18], we performed qRT-PCR to detect the expression of UCA1 after TGF-β1 treatment. Interestingly, the results showed that UCA1 was both significantly upregulated by TGF-β1 in the two cell lines (Figure 4D). Together, these results suggested that UCA1 could promote GBC cell metastasis and potentiate the epithelial cells to transdifferentiate into mesenchymal cells *in vitro*.

### UCA1 epigenetically repressed p21 and E-cadherin transcription via binding with EZH2 in GBC

**Figure 5:**
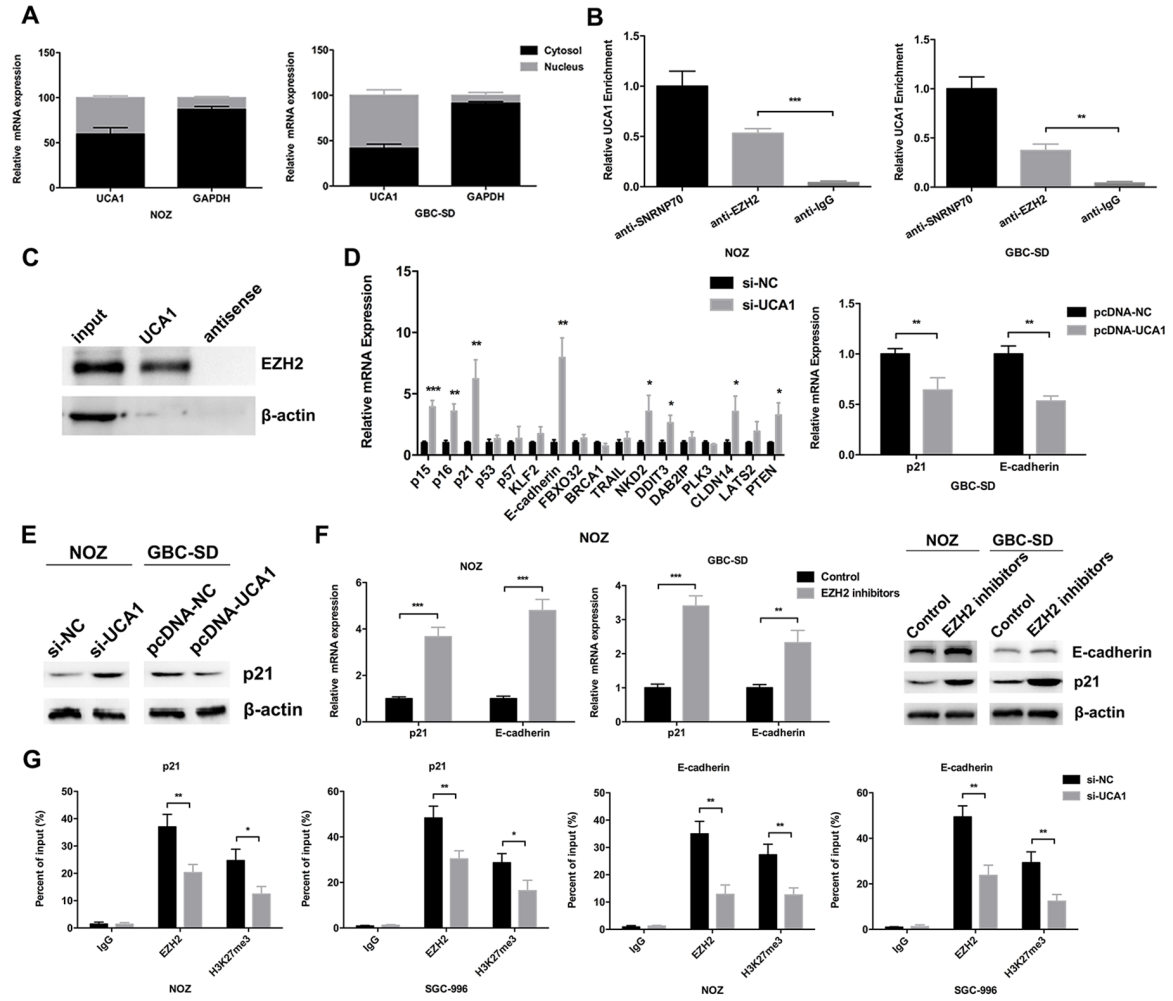
UCA1 bound with EZH2 to epigenetically repress p21 and E-cadherin transcription **(A)** Relative expression of UCA1 in cell cytoplasm or nucleus of NOZ and GBC-SD cells was detected by qRT-PCR. **(B)** Amount of UCA1 bound to SNRNP70 (a positive control), EZH2 or IgG (a negative control) was detected by qRT-PCR after RIP in NOZ and GBC-SD cells. **(C)** RNA pull-down assay was conducted using biotin-labeled UCA1 probe and detected the EZH2 expression by western blot assay. Antisense of the UCA1 probe was used as negative control. **(D)** Relative expression of p15, p16, p21, p53, p57, KLF2, E-cadherin, FBXO32, BRCA1, TRAIL, NKD2, DDIT3, DAB2IP, PLK3, CLDN14, LATS2 and PTEN mRNA in si-UCA1-transfected NOZ cells, relative expression of p21 and E-cadherin mRNA in pcDNA-UCA1-transfected GBC-SD cells was determined by qRT-PCR. **(E)** The protein levels of p21 in si-UCA1-transfected NOZ cells, or pcDNA-UCA1-transfected GBC-SD cells were determined by western blot assay. **(F)** Relative expression of p21 and E-cadherin mRNA, protein levels of p21 and E-cadherin in EZH2 inhibitors treated NOZ and GBC-SD cells were determined by qRT-PCR and western blot assay. **(G)** ChIP-qRT-PCR analysis of EZH2 occupancy, H3K27me3 binding to the p21 or E-cadherin promoter regions in NOZ and SGC-996 cells, and IgG as a negative control. The mean ± SD of triplicate experiments were plotted, *p<0.05, **p<0.01, ***p<0.001.

To explore the molecular mechanism of UCA1 in GBC, we first detected the distribution of UCA1 in two GBC cell lines (NOZ and GBC-SD). As shown in Figure 5A, UCA1 was both present in cytoplasm and nucleus of NOZ and GBC-SD cells. Since the “sponge” role of UCA1 in cytoplasm had been widely reported, in the present study, we aimed to explore whether UCA1 was physically associated with EZH2 that regulated targets at transcriptional level in nucleus. Next, RIP assay in NOZ and GBC-SD cells revealed that UCA1 was significantly enriched in the EZH2 antibody compared to the IgG (Figure 5B). To further confirm the direct binding between UCA1 and EZH2, we performed RNA pull down assay as well. There are three isoforms of UCA1 including 1.4, 2.2 and 2.7kb. Since the most abundant distribution of the 1.4kb isoform of UCA1, we focused on its functional significance in this study. The RNA pull down assay showed that biotin-labeled UCA1 could harbor EZH2 but not β-actin, which suggested that UCA1 could specially bind with EZH2 (Figure 5C).

Then we selected several EZH2 potential targets (p15, p16, p21, p53, p57, KLF2, E-cadherin, FBXO32, BRCA1, TRAIL, NKD2, DDIT3, DAB2IP, PLK3, CLDN14, LATS2, PTEN) with tumor-suppressor function that had been reported before, and postulated that they might be related to the contributions of UCA1 to GBC progression. The qRT-PCR results showed that the transcript levels of E-cadherin and p21 were increased most in UCA1 knockdown NOZ cells (Figure 5D). And the decreased transcript levels of E-cadherin and p21 could be observed in UCA1 overexpression GBC-SD cells (Figure 5D). Furthermore, the western blot assays confirmed these results in the protein level (Figure 5E). In addition, qRT-PCR and western blot results both showed that EZH2 specific inhibitors (EZP005687) led to increased p21 and E-cadherin expression in NOZ and GBC-SD cells (Figure 5F).

To validate whether UCA1 suppressed p21 and E-cadherin transcription through recruiting EZH2 to p21 and E-cadherin promoters, we performed ChIP assays in NOZ and SGC-996 cells and showed that EZH2 could directly bind to the promoters of p21 and E-cadherin and induce H3K27me3 trimethylation (Figure 5G). Moreover, UCA1 knockdown significantly reduced EZH2 binding and H3K27me3 trimethylation in NOZ and SGC-996 cells (Figure 5G). These results indicated that UCA1 contributes to GBC cell proliferation and metastasis, at least partly, through repressing p21 and E-cadherin expression.

### Repression of p21 is potentially involved in the oncogenic function of UCA1

**Figure 6:**
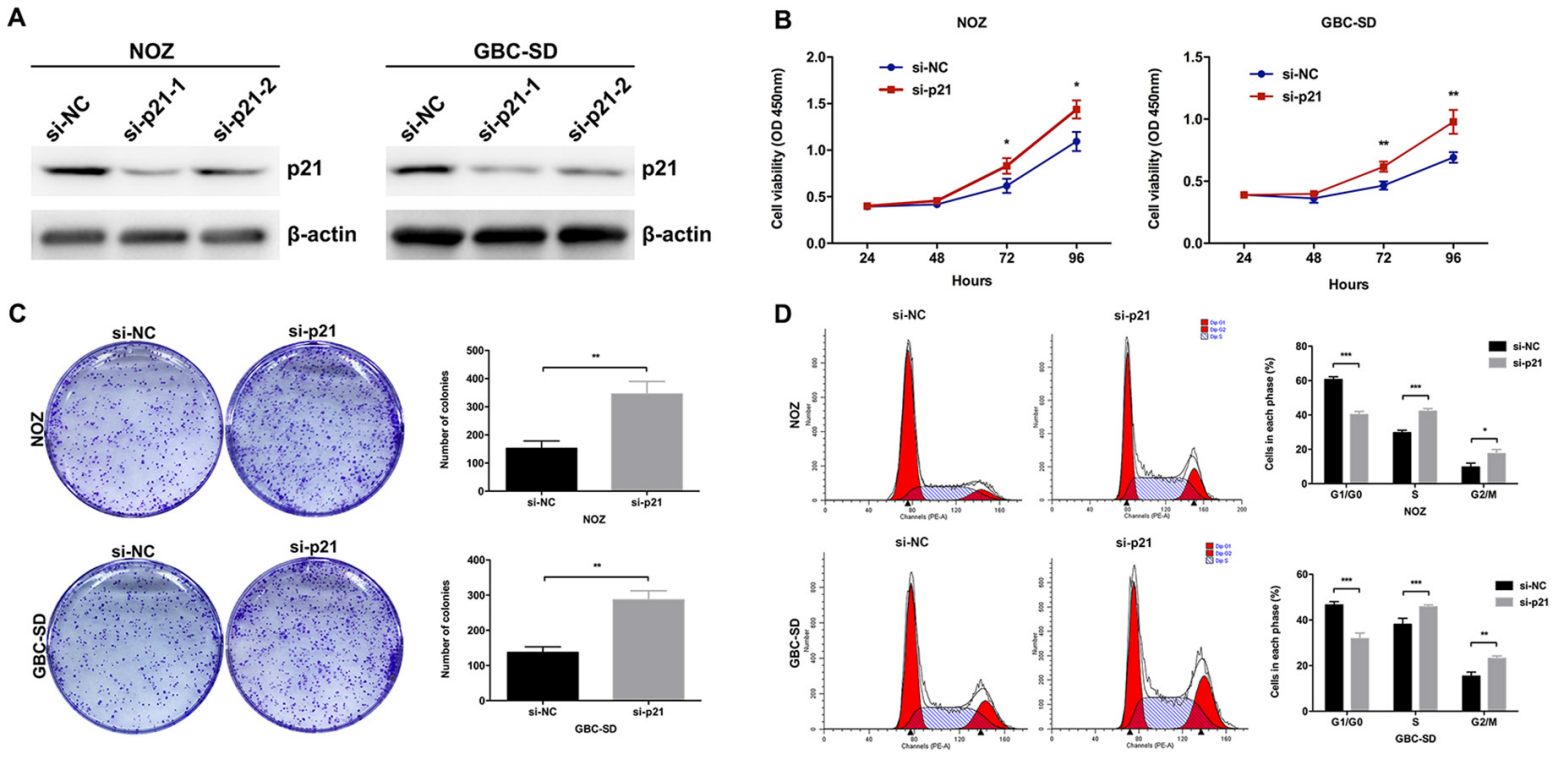
Effect of p21 on GBC cell growth *in vitro* **(A)** The protein levels of p21 in si-p21-transfected NOZ and GBC-SD cells were determined by western blot assay. **(B)** The cell viability of si-p21-transfected NOZ and GBC-SD cells was determined by CCK8 assays. **(C)** The coloning ability of si-p21-transfected NOZ and GBC-SD cells was determined by colony formation assays. **(D)** Flow cytometric analyses were performed to determine the cell cycle progression in si-p21-transfected NOZ and GBC-SD cells. The mean ± SD of triplicate experiments were plotted, *p<0.05, **p<0.01, ***p<0.001.

**Figure 7:**
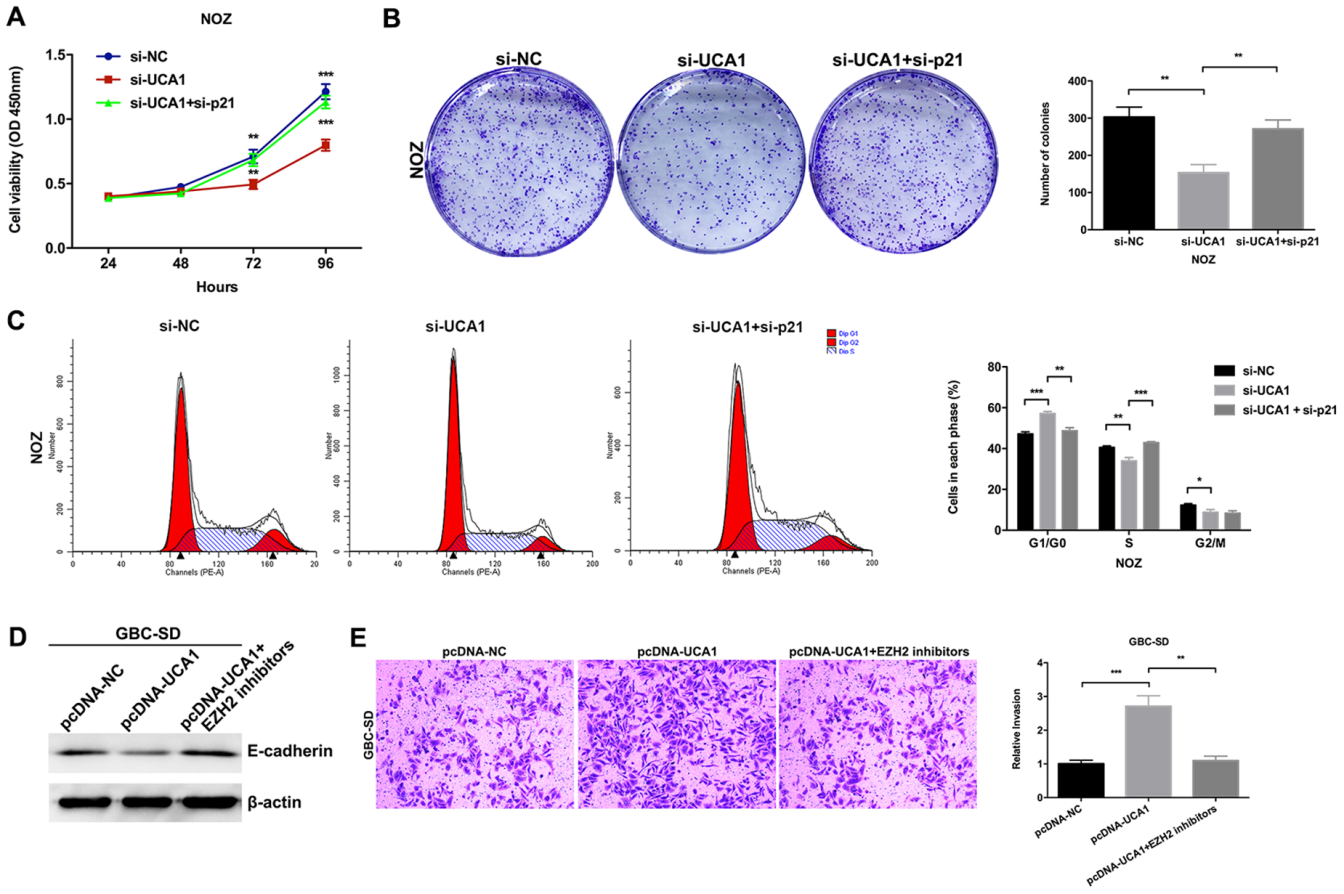
Effect of UCA1/EZH2/p21 and UCA1/EZH2/E-cadherin axes on GBC progression **(A)** The cell viability of si-NC-, si-UCA1- or si-UCA1 and si-p21-co-transfected NOZ cells was determined by CCK8 assays. **(B)** The coloning ability of si-NC-, si-UCA1- or si-UCA1 and si-p21-co-transfected NOZ cells was determined by colony formation assays. **(C)** Flow cytometric analyses were performed to determine the cell cycle progression in si-NC-, si-UCA1- or si-UCA1 and si-p21-co-transfected NOZ cells. **(D)** The protein levels of E-cadherin in pcDNA-NC-, pcDNA-UCA1- or EZH2 inhibitors treated and pcDNA-UCA1-transfected GBC-SD cells were determined by western blot assay. **(E)** The cell invasion ability in pcDNA-NC-, pcDNA-UCA1- or EZH2 inhibitors treated and pcDNA-UCA1-transfected GBC-SD cells was determined by transwell invasion assay. The mean ± SD of triplicate experiments were plotted, *p<0.05, **p<0.01, ***p<0.001.

Although the inhibitory effect of p21 on cell proliferation had been validated in other cancers, however, the role of p21 in GBC remained unclear. Then we designed two different p21 siRNAs and transfected them into NOZ cells. As shown in Figure 6A, the expression of p21 was more effectively knocked down by si-p21-1. The results of CCK8 and colony formation assays indicated that knockdown of p21 could significantly promote the NOZ cells proliferation (Figure 6B and 6C). The results of flow cytometric analysis indicated that knockdown of p21 could decrease NOZ cells G1-phase arrest (Figure 6D). These data indicated the functional role of p21 in GBC cell proliferation. Moreover, we performed rescue experiments to investigate the role of UCA1/EZH2/p21 axis in GBC cell proliferation. We co-transfected NOZ cells with UCA1 siRNAs and p21 siRNAs and found that the co-transfection could rescue the cell proliferation inhibition by the knockdown of UCA1 in NOZ cells (Figure 7A, 7B and 7C). While, the E-cadherin protein reduction and metastasis promotion by the overexpression of UCA1 in GBC-SD cells were rescued by the treatment of EZH2 inhibitors (Figure 7D and 7E).

## DISCUSSION

In the present study, we put the spotlight on the role of lncRNA UCA1 in GBC progression and demonstrated its significant upregulation in GBC patient tissues. Statistical analysis in clinical and pathologic characteristics suggested that the high expression level of UCA1 was significantly associated with GBC patients’ tumor size, lymph node metastasis, TNM stage and overall survival. Functionally, knocking down UCA1 in GBC cell inhibited cell proliferation, metastasis and EMT. By amplifying UCA1 in GBC cell, we observed the opposite phenomenon. Furthermore, the tumor xenograft experiment confirmed that UCA1 amplification could promote tumor growth *in vivo*. These results above indicated the oncogenic role of UCA1 in GBC. However, the corresponding mechanisms that contributed to this were remained unclear.

Through interacting with specific RNA-binding proteins, lncRNAs could activate the oncogenes or inactivate the tumor suppressors and then contribute to the proliferation or metastasis phenotype of cancer cells [7, 11]. EZH2, the crucial catalytic subunit of PRC2, is a histone methyltransferase that is specifically responsible for H3K27me3 trimethylation of target genes and represses their expression [21]. As reported, approximately 20% of all human lncRNAs identified are physically associated with EZH2 [22, 23]. To explore the relationship between UCA1 and EZH2 in GBC, in this study, we performed RIP and RNA pull-down assays and demonstrated the direct interaction between UCA1 and EZH2. ChIP assay showed that EZH2 could bind to the promoter regions of p21 and E-cadherin, which was also confirmed by Xie’ study and Sun’ study [23, 24]. Additionally, after knocking down the expression of UCA1, we found that the binding of EZH2 to p21 and E-cadherin was decreased. After comprehensive consideration, we put forward for the first time that UCA1 might exert oncogenic effects on GBC progression by recruiting EZH2, which binds to the promoter of p21 and E-cadherin to repress their transcript.

As is well known, cell proliferation is closely related to the cell cycle, and loss of cell cycle control partly contributed to tumorigenesis, including GBC. The mammalian cell cycle progression is manipulated by the regulatory subunits cyclins and cyclin-dependent kinases (CDKs) [25]. P21 (p21^waf1/cip1^ or p21/CDKN1A), an important CDKs inhibitor family member, can arrest the cell cycle progression in G1/S transition by disrupting the interaction between CDKs and cyclins [26, 27]. Li et al. [28] performed immunohistochemistry in GBC tissues and demonstrated that the expression of p21 was frequently decreased and reduced p21 was significantly associated with shortened disease-free and overall survival for patients with stages II to IV GBC patients. However, in the present study, we first confirmed the function of p21 in GBC cell proliferation *in vitro* and the rescue experiments showed that UCA1-mediated tumor promoting effects on GBC cell was partly dependent on the epigenetic silencing of p21 expression. While, we also found a similar regulatory manner of UCA1 on the major epithelial marker E-cadherin. E-cadherin, a tumor suppressor in cancer development, is regulated by multiple enzymes involving epigenetic modifications [29]. The loss of E-cadherin increases tumor cell migration and invasion, and leads to tumor dissemination [30]. Some identified EMT-inducing transcription factors (Snail, Slug, ZEB1, ZEB2, Twist1, Twist2, etc.) could silence the transcription of E-cadherin by directly binding to the E-box motifs of E-cadherin promoter and recruiting multiple corepressors to this region [31–33]. Additionally, TGF-β1 is an acknowledged EMT inducer that changes fibroblast growth characteristics. Zuo et al. reported that UCA1 promoted gastric cancer invasion and metastasis under TGF-β1 induction [34]. Our present study found a similar phenomenon in GBC and partly explained the mechanism by which UCA1 promoted GBC cell EMT and then influenced the metastasis.

Collectively, we identified that lncRNA UCA1 promoted GBC progression by recruiting EZH2 to the promoters of tumor suppressors p21 and E-cadherin, and consequently decelerated their transcript. Our findings not only highlighted the role of UCA1 in GBC progression, but also revealed a new axis by which UCA1 promoted GBC cell proliferation and metastasis. Along with further research, UCA1 might be a prognostic indicator as well as a therapeutic target for GBC.

## MATERIALS AND METHODS

### Patients and tissue samples

This study was approved by the Human Ethics Committee of Xinhua Hospital (Shanghai JiaoTong University School of Medicine, Shanghai, China). A cohort of forty-five GBC tissues and neighboring noncancerous tissues were obtained from patients who underwent liver resection from November 2009 to October 2012 in Xinhua Hospital (Shanghai JiaoTong University School of Medicine, Shanghai, China) and Eastern Hepatobiliary Surgery Hospital (Second Military Medical University, Shanghai, China). Informed consent was obtained from all patients. All patients were diagnosed with GBC according to two independent pathologists’ evaluation. There was no any pre-operative treatment conducted in the recruited patients.

### Cell culture, qRT-PCR, western blot, cell transfection, cell invasion assay, cell proliferation assay, flow cytometric analysis, chromatin immunoprecipitation (ChIP) assay and immunohistochemical staining

Cell culture, qRT-PCR, western blot, cell transfection, cell invasion assay, cell proliferation assay, flow cytometric analysis, ChIP assay and immunohistochemical staining were performed as described previously [9]. The antibodies for western blot were anti-p21 (1:2000, Proteintech, China), anti-E-cadherin (1:5000, Proteintech, China), anti-Vimentin (1:5000, Proteintech, China) and anti-β-actin (1:5000, Proteintech, China). The antibodies for ChIP were anti-EZH2 (1:100, Cell Signaling Technology, USA) and anti-H3K27me3 (1:50, Cell Signaling Technology, USA). The antibody for immunohistochemical staining was anti-Ki-67 (1:200, Cell Signaling Technology, USA). Vectors pcDNA-UCA1 and LV-GFP-UCA1 were brought from Genechem (Shanghai, China). Primers for qRT-PCR, siRNAs sequence and ChIP are shown in Supplementary Table 1.

### Wound healing assay, RNA-binding protein immunoprecipitation assay (RIP) and immunofluorescence analysis

Wound healing assay, RNA-binding protein immunoprecipitation assay (RIP) and immunofluorescence analysis were performed as described previously [6]. The antibodies for RIP were anti-EZH2 (1:50, Cell Signaling Technology, USA). The antibodies for immunofluorescence analysis were anti-E-cadherin (1:200, Proteintech, China), anti-Vimentin (1:200, Proteintech, China). Primers for RIP are shown in Supplementary Table 1.

### Colony formation assay

Approximately 1000 transfected NOZ or GBC-SD cells were seeded into each well of 6-well plates and cultured in media with 10% fetal bovine serum. After two weeks, cells were treated with methanol and stained with 0.1% crystal violet. The number of visible colonies was counted.

### Ethynyldeoxyuridine (EdU) retention assay

The EdU labeling/detection kit (Ribobio, China) was used according to the manufacturer's instructions to evaluate cell proliferation. Forty-eight hours after transfection, approximately 5000 transfected NOZ cells were seeded into each well of 96-well plates, 50 μM EdU labeling media was added and incubated for 2 hours at 37°C under 5% CO_2_. Then cells were treated with 4% paraformaldehyde, 0.5% Triton X-100 and anti-EdU working solution successively. The percentage of EdU-positive cells was calculated using fluorescent microscopy.

### Subcellular fractionation

To determine the cellular localization of UCA1, cytoplasm and nuclear fractions were isolated and collected with RNeasy Midi Kit (Qiagen, Germany) according to the manufacturer's instructions. RNAs extracted from each of the fractions were subjected to following qRT-PCR analysis of the levels of GAPDH and UCA1.

### RNA pull-down assay

RNA pull-down assay was performed using Magnetic RNA-Protein Pull-Down Kit (Pierce, USA) according to the manufacturer's instructions. First, the full length of UCA1 was synthesized using RiboMAX™ Large Scale RNA Production Systems (Promega, USA). After biotin labeling, UCA1 was bound to the beads for protein binding. Cell protein lysate was added with RNA-bound beads for immunoprecipitation. Beads were washed three times and boiled in SDS buffer, and the retrieved protein was detected by western blot analysis. The antibody for RNA pull-down assay was anti-EZH2 (1:1000, Cell Signaling Technology, USA).

### Tumor xenograft experiment

Each 4-week-old male nude mouse (five mice per group) was subcutaneously injected with GBC-SD cells (100μl, 1 × 10^6^) that stably expressing LV-UCA1 or LV-NC. Tumor volumes were calculated as 0.5 × length × width^2^ on a weekly basis. After four weeks, mice were sacrificed, and tumors were excised, weighed and subjected to immunofluorescence analysis for Ki-67 expression. All animal experiments were performed in animal laboratory center of Xinhua Hospital (Shanghai JiaoTong University School of Medicine, Shanghai, China). The study protocol was approved by the Animal Care and Use committee of Xinhua Hospital.

### Statistical analysis

Statistical analysis was performed using SPSS 20.0 (SPSS, USA). Data were presented as mean ± standard deviation (SD). Paired samples *t*-test was used to analyze the expression differences of UCA1 between GBC tissues and neighboring noncancerous tissues. Independent samples *t*-test was used to analyze the differences between groups. Kaplan-Meier method was used to analyze the survival, and log-rank test was used to determine the significance. Pearson's correlation coefficient was applied for expression correlation assay. *P* values were two-side and a *p* value less than 0.05 was considered to be statistically significant.

## SUPPLEMENTARY MATERIALS AND TABLES





## Abbreviations

LncRNAs: long non-coding RNAs
UCA1: urothelial carcinoma associated 1
GBC: gallbladder cancer
qRT-PCR: quantitative real-time polymerase chain reaction
CCK8: cell counting kit-8
EdU: ethynyldeoxyuridine
RIP: RNA-binding protein immunoprecipitation
ChIP: chromatin immunoprecipitation
EZH2: enhancer of zeste homolog 2
miRNAs: microRNAs
H3K27me3: histone H3 at lysine 27 trimethylation
SD: standard deviation
EMT: epithelial-mesenchymal transition
PRC2: polycomb repressive complex 2.
