# Supplementary material for: Long non-coding RNA UCA1 promotes gallbladder cancer progression by epigenetically repressing p21 and E-cadherin expression
Source: Oncotarget. 2017 May 25;8(29):47957–68. doi: 10.18632/oncotarget.18204 (PMC5564618; doi:10.18632/oncotarget.18204)
Supplement: Supplementary file 1 [file oncotarget-08-47957-s001.pdf]

Long non-coding RNA UCA1 promotes gallbladder cancer progression by epigenetically repressing p21 and E-cadherin expression

SUPPLEMENTARY INFORMATION

Supplementary Table 1: Information of the qRT-PCR primer sequence and siRNA sequence

See Supplementary File 1
